# Prediction models for short children born small for gestational age (SGA) covering the total growth phase. Analyses based on data from KIGS (Pfizer International Growth Database)

**DOI:** 10.1186/1472-6947-11-38

**Published:** 2011-06-01

**Authors:** Michael B Ranke, Anders Lindberg, KIGS International Board

**Affiliations:** 1 Paediatric Endocrinology Section, Children's Hospital, University of Tuebingen, D-72076 Tuebingen, Germany; 2 Pfizer Inc., Pfizer Endocrine Care, KIGS/KIMS/ACROSTUDY Medical Outcomes SE-191 90 Sollentuna, Sweden

## Abstract

**Background:**

Mathematical models can be developed to predict growth in short children treated with growth hormone (GH). These models can serve to optimize and individualize treatment in terms of height outcomes and costs. The aims of this study were to compile existing prediction models for short children born SGA (SGA), to develop new models and to validate the algorithms.

**Methods:**

Existing models to predict height velocity (HV) for the first two and the fourth prepubertal years and during total pubertal growth (TPG) on GH were applied to SGA children from the KIGS (Pfizer International Growth Database) - 1^st ^year: N = 2340; 2^nd ^year: N = 1358; 4^th ^year: N = 182; TPG: N = 59. A new prediction model was developed for the 3^rd ^prepubertal year based upon 317 children by means of the all-possible regression approach, using Mallow's C(p) criterion.

**Results:**

The comparison between the observed and predicted height velocity showed no significant difference when the existing prediction models were applied to new cohorts. A model for predicting HV during the 3^rd ^year explained 33% of the variability with an error SD of 1.0 cm/year. The predictors were (in order of importance): HV previous year; chronological age; weight SDS; mid-parent height SDS and GH dose.

**Conclusions:**

Models to predict growth to GH from prepubertal years to adult height are available for short children born SGA. The models utilize easily accessible predictors and are accurate. The overall explained variability in SGA is relatively low, due to the heterogeneity of the disorder. The models can be used to provide patients with a realistic expectation of treatment, and may help to identify compliance problems or other underlying causes of treatment failure.

## Background

Small for gestational age (SGA) is a working diagnostic term used to describe foetuses or newborn infants who have a lower weight and/or length than what is normal for their gestational age, in the absence of any other specific diagnosis or reason for their small stature [1]. Although in the majority of children born SGA catch-up growth occurs by 2 years of age, in about 10% catch-up growth does not occur. Without treatment, these children remain short and constitute some 20-25% of adults whose final height is below -2 SD scores [2]. Although, these individuals are not growth hormone (GH) deficient, recent long-term studies have shown that treatment with recombinant human GH is able to improve height. Today GH has become an approved treatment in promoting catch-up growth [3] in short children born SGA. Experience with SGA children treated with GH to adult height is still very limited and studies have shown that a considerable fraction of the children treated does not achieve an adult height within the normal range. These observations demand an improved and individualized therapy with GH in short children with SGA. Recently, growth prediction models for the optimization of GH in SGA have been published based on patients documented within KIGS [4,5]. The aim of this study is to test the existing prediction models and to develop additional growth prediction model from the KIGS database, to allow an optimisation and individualization of GH treatment from pre-pubertal onset to the end of the growth phase in SGA.

## Methods

### Patients

Patients included in this analysis were receiving recombinant human GH (Genotropin^®^; Pfizer Inc.) during follow-up and were documented in the pharmacoepidemiological survey - KIGS (Pfizer International Growth Database) by July 18, 2010. The KIGS survey is conducted in accordance with the Declaration of Helsinki [6]. Diagnosis was made according to the KIGS aetiology classification list [7]. Additional inclusion criteria for the patients were a birth weight and/or birth length for gestational age below -2.0 SD scores. Pre-pubertal stage was defined as a mean testes volume ≤ 3 ml in boys or Tanner breast stage B1 in girls. All patients received six or seven injections of GH per week. In order to avoid the effects of initial catch-up growth on total pubertal growth (TPG) only patients who were treated with GH for a total of at least 5 years and who were treated at least two years before puberty onset were included in the analysis of TPG.

The height standards used for normal children were those of Tanner et al [8] or - as indicated - those of Prader et al. [9] and the weight standards were those of Freeman et al. [10]. Birth weight for gestational age was transformed to an SD score based on the standards of Niklasson et al [11]. The MPH SD score was calculated as: (father's height SD score + mother's height SD score) ÷ 1.61 [12]. Bone ages, calculated according to the method of Greulich and Pyle [13], were taken as reported by the treating physician.

The existing prediction models for the first two prepubertal years for short children born SGA [4] were tested on children documented within KIGS by July 18, 2010. The characteristics of the patients used for the development of prediction models for the first two prepubertal years are listed in Table 1. The existing prediction models for total pubertal growth (TPG) developed for boys and girls with idiopathic GHD [5] were tested for applicability to SGA children.

**Table 1:** Characteristics of the SGA patients used for the development of the SGA growth prediction models

	1^st ^prepubertal year			2^nd ^prepubertal year		
	**N**	Mean	SD	**N**	Mean	SD

At GH treatment start						
Sex male %		67			62	
Birth weight SDS	613	-2.7	1.0	385	-2.6	1.0
Birth length SDS	465	-2.7	1.3	295	-2.7	1.3
Maximum GH peak (ng/ml)	613	20.8	13.3	385	20.9	13.6
MPH SDS	607	-0.9*	1.2	379	-0.8*	1.2
Age (years)	613	6.6	2.5	385	6.3	2.2
Height SDS	613	-2.8*	0.9	385	-2.8*	0.9
Weight SDS	613	-3.4	1.6	385	-3.4	1.6
GH dose (mg/kg/day)	613	0.04	0.02	385	0.04	0.02
First-year growth response						
HV(cm/year)	613	8.6	1.9	385	8.7	1.8
Change in (delta) height SDS	613	0.7*	0.4	385	0.7*	0.3
Second year growth response						
HV(cm/year)				385	7.0	1.4
Change in (delta) height SDS				385	0.3*	0.2

The first and second pre-pubertal years on GH [4] are shown.

* = calculations based on Tanner's references [8]

### Development of the prediction model

Growth responses (annualized height velocities) during GH therapy were correlated, by multiple regression analysis, with potentially relevant variables as published before [14]. It was attempted to develop prediction models for the third and fourth pre-pubertal year on GH. The variables tested were (i) status at birth: sex, weight SD score, length SD score, ponderal index, mode of delivery and APGAR score; (ii) genetic background: height SD score of the mother, height SD score of the father, and midparental height (MPH) SD score; (iii) treatment modality: GH dose (per kg body weight and per kg ideal body weight [weight for height]), frequency of GH injections, and accumulated years of GH treatment; (iv) patient variables at the beginning of the treatment period: age, bone age, height SD score, weight SD score, height SD score minus MPH SD score, the peak serum GH concentration during stimulation testing and the height velocity during the previous year of GH treatment.

### Statistical analysis

Variables are reported as mean and standard deviation. SD scores were calculated as follows: SD score = (patient value - mean value for age- and sex-matched normal subjects) ÷ SD of the value for age- and sex-matched normal subjects. For comparison of patient groups, Student's t-test was used if data had a Gaussian distribution, and Wilcoxon's rank-sum test was used otherwise. Significance was considered at the 1% level (P < 0.01), unless otherwise specified. SAS^® ^version 8 for Sun Solarix (SAS Institute, Cary, North Carolina) was used for all statistical analyses. The prediction models were developed by means of a multiple linear regression analysis fitted by least squares and the REG procedure in the SAS computer program (version 8.02, SAS Institute, Inc., Cary, NC). A hierarchy of predictive factors was derived by the all-possible regression approach, using Mallow's C (p) criterion for ordering predictive factors, as described previously [15,16]. Differences between observed and predicted height velocities were expressed in terms of Studentized Residuals. The residual is calculated as the observed height velocity minus the predicted height velocity for each observation, and the Studentized Residual is the residual divided by its standard error, which is equivalent to a SD score.

## Results

### Response to GH: 1^st ^prepubertal year

A total of 2340 short children (62% boys) born SGA were treated a full first year with GH. The characteristics at start of GH treatment and the responses to GH are listed in Table 2. The characteristics of the patients and the parameters relevant for the developed first-year prediction model [4] are listed in Table 1. The patients were started on GH at an age of 7.2 (+/- 3.2) years and a height of -3.4 (+/- 1.0) SDS. The height velocity was 8.5 (+/- 2.1) cm/year and the delta height was 0.75 (+/- 0.44) SDS. The predicted height velocity was 8.8 (+/- 1.1) cm/year, which resulted in an SR of -0.27 (+/- 1.3 (see Table 2).

**Table 2:** Characteristics of the SGA patients used for the present analysis

	1^st ^pre-pubertal year			2^nd ^pre-pubertal year			3^rd ^pre-pubertal year			4^th ^pre-pubertal year		
	N	Mean	SD	N	Mean	SD	N	Mean	SD	N	Mean	SD

At GH treatment start												
Sex male %	2340	62		1358	65		317	72		182	62	
Birth weight SDS	2339	-2.6	1.0	1357	-2.6	1.0	316	-2.6	1.0	181	-2.8	1.0
Birth length SDS	1927	-2.5	1.3	1132	-2.6	1.3	254	-2.8	1.4	155	-3.1	1.4
Maximum GH peak (ng/ml)	1324	19.5	13.6	804	20.2	14.6	221	19.2	14.4	116	21.2	15.4
MPH SDS	2157	-1.2	1.2	1253	-1.1	1.2	317	-0.9	1.3	167	-0.7	1.3
Age (years)	2340	7.2	3.1	1358	6.7	2.8	315	5.5	2.1	182	3.7	0.9
Height SDS	2340	-3.4	1.0	1358	-3.5	1.0	317	-3.7	1.1	182	-4.2	1.3
Weight SDS	2340	-3.1	1.5	1358	-3.2	1.5	317	-3.6	1.7	182	-4.2	1.9
HV(cm/year)	756	5.4	1.9	486	5.5	1.9	140	5.6	3.7	88	6.5	2.1
GH dose (mg/kg/week)	2340	0.30	0.10	1358	0.31	0.11	317	0.30	0.11	182	0.31	0.11
First-year growth response												
HV (cm/year)	2340	8.5	2.1	1358	8.7	2.0	310	9.0	2.0	173	9.5	2.0
Change in (delta) height SDS	2340	0.75	0.44	1358	0.79	0.43	310	0.82	0.43	173	1.0	0.5
Second year growth response												
HV(cm/year)				1358	7.2	1.5	317	7.4	1.4	174	7.9	1.5
Change in (delta) height SDS				1358	0.45	0.28	310	0.47	0.27	174	0.6	0.3
Third year growth response												
HV(cm/year)							317	6.6	1.2	182	6.9	1.4
Change in (delta) height SDS							317	0.32	0.22	182	0.31	0.29
Fourth year growth response												
HV(cm/year)										182	6.1	1.2
Change in (delta) height SDS										182	0.20	0.25
Observed vs. predicted	**1^st ^yr**			**2^nd ^yr**			**3^rd ^yr**			**4^th ^yr**		
Predicted HV(cm/yr)	1895	8.84	1.13	1104	7.08	0.67	317	6.60	0.71	171	6.19	0.78
Observed HV (cm/yr)	1895	8.48	1.90	1104	7.13	1.45	317	6.60	1.24	171	6.10	1.17
Student. Residual (SDS)	1895	-0.27	1.29	1104	0.04	1.22	317	0.00	1.01	171	-0.10	1.19

1st, 2nd, 3rd and 4th prepubertal years and the comparison between observed and predicted height velocities within respective years are shown.

Calculated derivates of height data (SDS, MPH) are based on Prader's references. [9]

### Response to GH: 2^nd ^prepubertal year

A total of 1358 short stature children (65% boys) born SGA were treated a full second year with GH. The characteristics at start of GH treatment and the responses to GH are listed in Table 2. The characteristics of the patients and the parameters relevant for the developed second-year prediction model [4] are listed in Table 1. The patients were started on GH at an age of 6.7 (+/- 2.8) years and a height of -3.5 (+/- 1.0) SDS. The height velocity during the second year was 7.2 (+/- 1.5) cm/year and the delta height was 0.45 (+/- 0.28) SDS. The predicted height velocity was 7.1 (+/- 0.7) cm/year, which resulted in an SR of 0.04 (+/- 1.2).

### Response to GH: 3^rd ^prepubertal year

A total of 317 short stature children (72% boys) born SGA were treated a full third year with GH. The characteristics at start of GH treatment and the responses to GH are listed in Table 2. The patients were started on GH at an age of 5.5 (+/- 2.1) years and a height of -3.7 (+/- 1.1) SDS. The height velocity during the third year was 6.6 (+/- 1.2) cm/year and the delta height was 0.32 (+/- 0.22) SDS. The group was used to develop a third-year prediction model for SGA (see below).

### Response to GH: 4th prepubertal year

A total of 182 short stature children (62% boys) born SGA were treated a full fourth year with GH. The characteristics at start of GH treatment and the responses to GH are listed in Table 2. The patients were started on GH at an age of 3.7 (+/- 0.9) years and a height of -4.2 (+/- 1.3) SDS. The height velocity during the fourth year was 6.1 (+/- 1.2) cm/year and the delta height was 0.20 (+/- 0.25) SDS. The predicted height velocity based on the fourth year prediction model of GHD patients [14] was 6.2 (+/- 0.8) cm/year, which resulted in an SR of -0.10 (+/- 1.2).

### Response to GH: Total pubertal growth (TPG)

In 35 male patients whose puberty started at an age of 12.9 (+/- 1.1) years and a height of 140.1 (+/- 6.8) cm, and who received a mean GH dose of 0.26 (+/-0.1) mg/kg/week TPG was 23.0 (+/-6.2) cm. In 24 female patients whose puberty started at an age of 11.8 (+/- 1.8) years and a height of 135.4 (+/- 7.2) cm, and who received a mean GH dose of 0.25 (+/-0.1) mg/kg/week TPG was 17.3 (+/-6.3) cm. Based on the prediction models developed for TPG of males and females with GHD [17] the predicted TPG for boys and girls were 26.3 (+/-5.1) and 18.0 (+/-5.7) cm. These predicted numbers for height gain during puberty are not different from those actually observed.

### 3^rd ^year prepubertal prediction model for short children born SGA

The results of the development of the prediction model for the 3^rd ^prepubertal year based on 317 children (see Table 2) are listed in Table 3. Table 3 also gives the rank order of importance of the variables as predictors, the overall correlation coefficients of the prediction models (R^2^) and the error SD of the prediction in centimetres. The equation describing the predicted height velocity (PHV) for the third year of GH therapy (from Table 3) is as follows: PHV (cm/year) = 6.2 + [-0.18 × age at start (years)] + [0.19 × weight SD score at start] + [1.21 × GH dose (mg/kg/week)] + [0.12 × MPH SD score] + [0.26 × HV 2^nd ^year (cm/year)] ± 1.0. The equation explains 33% of the variability.

**Table 3:** Regression equation variables for prediction of the growth response to GH therapy during various treatment periods in short children born SGA

	Prepubertal years								Total pubertal growth				
	1^st ^year		2^nd ^year		3^rd ^year		4^th ^year ^(@)^			male ^(@)^		female ^(@)^	

Parameter									Parameter				
N	613		385		317		180			355		221	
R^2^	0.52		0.34		0.33		0.30			0.66		0.65	
Error SD (cm)	1.3		1.1		1.0		1.0			4.5		3.8	
Parameter estimate (PE)	PE	Rank	PE	Rank	PE	Rank	PE	Rank		PE	Rank	PE	Rank
Intercept (constant)	9.4		4.7		6.2		6.0		Intercept	72.6		57.0	
Age at start (years)	-0.31	2	-0.11	2	-0.18	2	-0.05	4	Age puberty onset (years)	-4.0	1	-3.7	1
Weight at start (SDS)	0.30	3	-	-	0.19	3	0.40	1	Age - Bone age (years)	1.4	2	1.7	2
GH dose at year start (mg/kg/day)	56.51	1	13.46	3	1.21^#^	5	0.87^&^	3	Mean GH dose (mg/kg/week)	8.8	4	9.5	4
MPH (SDS)	0.11 *	4	-	-	0.12^$^	4	-	-	Height - MPH ^$^ (SDS)	-1.3	3	-1.2	3
HV in previous year (cm/year)	-	-	0.30	1	0.26	1	0.21	2	_	_	_	_	_

* = based on Tanner references; ^$ ^[8] = based on Prader references [9]; ^# ^= mg/kg/week; ^&^= ln IU/kg/week; ^@ ^= based on GHD model (Ranke et al., 1999) [17]

**Figure 1:**
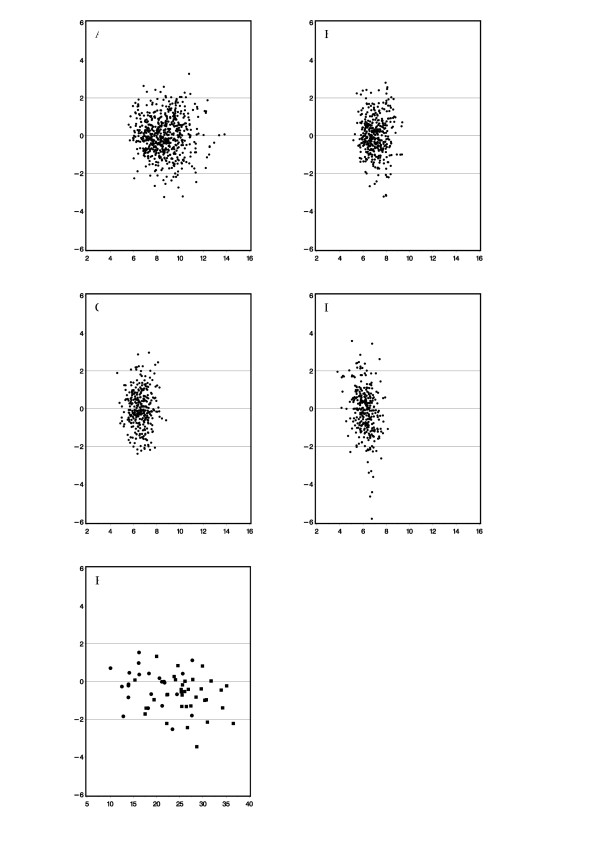
**Studentized Residuals (y-axis) in relationship to the predicted growth (x-axis)**. a) 1^st ^prepubertal year; b) 2^nd ^prepubertal year; c) 3^rd ^prepubertal year, d) 4^th ^prepubertal year, and e) TPG (closed circles = males; open circles = females); a-d: X-axis in cm/year; e X-axis in cm.

The difference between observed and predicted height velocity during the first to fourth prepubertal year and of observed and predicted total growth during puberty expressed in terms Studentized are illustrated in Figures 1 a-e.

### Validation of 3^rd ^year prepubertal prediction model for SGA children

**Figure 2:**
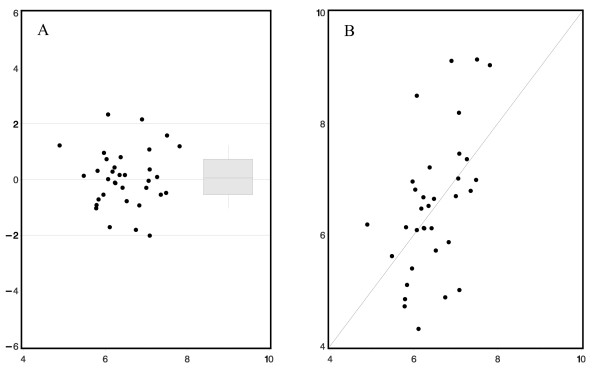
**Validation group (N = 34) for the 3^rd ^prepubertal year model: a) Studentized Residuals (y-axis) in relationship to the predicted growth (x-axis); b) correlation between predicted (x-axis) and observed (y-axis) height velocity (cm/year)**.

A subgroup of 34 children was randomly assigned from the total cohort identified for the validation of the derived model. The characteristics of these patients were not different compared to model group. The correlation between the predicted and the observed height velocity in the validation group was: R = 0.53 (p = 0.001) and the mean of Studentized Residual was 0.0 (SD = 1.0) [Figure 2a, b].

## Discussion

Most short stature children born SGA catch up within the first two years of life; however, about 10-15% remain permanently short [3]. Obviously, since the causes of intrauterine as well as permanent growth impairment are often unknown, the terminology used to define these children is descriptive rather than causal [1]. GH treatment in short children born SGA has been investigated over many years. Both the US Food and Drug Administration (FDA) and the European Agency for the Evaluation of Medical Products (EMEA) have approved GH for the treatment of short stature in children born SGA and Silver-Russell syndrome, provided that other causes of short stature are excluded, such as growth-inhibiting medication, chronic diseases, endocrine disorders, emotional deprivation, or syndromes associated with short stature. GH treatment for short stature in SGA children is standard practice today [18,19]. So far only a few studies on SGA have documented the growth response to GH until adult height [20-28]. The data suggest that a considerable fraction of the short stature children born SGA treated with GH does not reach a normal height. Thus, an optimising of the treatment approach with GH is mandatory.

The response to GH can be expressed in different modes. Traditionally the response has been expressed in terms of annual height velocity (cm/year) which can be compared to normal age-related references either directly or transformed into SD scores. During puberty total height gain (cm) from the onset of puberty to the end of growth can be described. In addition height velocity can be compared with empirical somatograms derived from patients during GH therapy versus age and sex. Such height velocity targets [29] have been published for the first two prepubertal years for SGA children [30]. Moreover, the response can be predicted based on mathematical algorithms derived from the analyses of large cohorts of children with the same diagnosis treated with GH [31]. The observed and the predicted growth can be compared and expressed as a Studentized Residual; - an" index of responsiveness".

We and others [32-34,17] ) have developed algorithms (models) for the prediction of the response to GH and have shown that the most important determinants of first pre-pubertal year growth on GH in short children born SGA including Silver Russell Syndrome (SRS) are the dose of GH (the higher, the greater) and the age of the children (the younger, the greater). We were also able to show that the height velocity observed during the first year is the major determinant of the second pre-pubertal year growth response to GH in SGA [4]. Unlike in GHD there are no SGA growth prediction models existing for the subsequent pre-pubertal treatment years. The present analysis confirms that the SGA prediction models developed for the first two prepubertal years are suited to accurately predict height velocity in other patients documented within KIGS, who by the nature of the database are very heterogeneous. During the third prepubertal year of GH treatment again the observed previous height velocity and age are the most important predictors of height velocity. During the fourth year of GH treatment in SGA growth is only marginally above the spontaneous normal growth rate. Thus, most of the catch-up growth occurs during the first three years on GH. Since the number of children during the fourth prepubertal treatment year within KIGS did not exceed two hundred it was not appropriate to calculate an independent prediction model for SGA. However, the fact that the fourth-year prediction model for children with GHD [14] could be applied to the SGA cohort indicates, that after most of catch-up growth has occurred, similar mechanisms appear to be involved in the response to GH of both disorders at that stage of treatment.

Likewise, the number of individuals with all documented information required for the development of a disease-specific mathematical algorithm to predict TPG was presently still too small within KIGS. However, the models developed for male and female adolescents with GHD [17] could be applied to adolescents with SGA and gave accurate results. The models for TPG show that the anthropometrical characteristics at puberty onset are more important for TPG than the dose of GH, which is of course the only factor which can be modified by the treating physician.

## Conclusions

The presently available growth prediction models derived from KIGS data are suited to predict height development during the most relevant phases of growth in short children born SGA. This will serve to adapt treatment modalities in such a way that an optimal height outcome can be achieved. The prediction models can be used to provide patients with a realistic expectation of treatment, and may help to identify compliance problems or other underlying causes of treatment failure.

## Competing interests

MBR is a consultant to KIGS and receives honoraria from Pfizer, NovoNordisk, Eli Lilly and Ipsen. MBR was a paid consultant to Pfizer in connection with the development of this manuscript.

AL is a full-time employee of Pfizer Endocrine Care, KIGS/KIMS/ACROSTUDY, Stockholm, Sweden

## Authors' contributions

MBR has made major contributions in designing the analyses and in interpretation of data, in drafting the manuscript and revising it critically for important intellectual content. AL participated in the design of the analyses and performed the statistical analyses. Both authors read and approved the final manuscript.

## Pre-publication history

The pre-publication history for this paper can be accessed here:

http://www.biomedcentral.com/1472-6947/11/38/prepub
